# Transcriptome and Gene Expression Analysis Revealed *CeNA1*: A Potential New Marker for Somatic Embryogenesis in Common Centaury (*Centaurium erythraea* Rafn.)

**DOI:** 10.3390/ijms252413531

**Published:** 2024-12-18

**Authors:** Katarina B. Ćuković, Slađana I. Todorović, Jelena M. Savić, Milica D. Bogdanović

**Affiliations:** Department of Plant Physiology, Institute for Biological Research “Siniša Stanković”—National Institute of Republic of Serbia, University of Belgrade, Bulevar Despota Stefana 142, 11108 Belgrade, Serbia; slatod@ibiss.bg.ac.rs (S.I.T.); savic.jelena@ibiss.bg.ac.rs (J.M.S.); milica.bogdanovic@ibiss.bg.ac.rs (M.D.B.)

**Keywords:** embryogenic callus, globular somatic embryo, cotyledonary somatic embryo, auxin-related genes, stress-related genes, differentially expressed genes

## Abstract

*Centaurium erythraea* Rafn. is a medicinal plant used as a model for studying plant developmental processes due to its developmental plasticity and ease of manipulation in vitro. Identifying the genes involved in its organogenesis and somatic embryogenesis (SE) is the first step toward unraveling the molecular mechanisms underlying its morphogenic plasticity. Although SE is the most common method of centaury regeneration, the genes involved in this have not yet been identified. The aim of this study was to identify the differentially expressed genes (DEGs) during key stages of SE and organogenesis using transcriptome data, with a focus on novel SE-related genes. The transcriptomic analysis revealed a total of 4040 DEGs during SE and 12,708 during organogenesis. Gene Ontology (GO) annotation showed that the highest number of SE-related genes was involved in defense responses. The expression of fifteen selected SE-related candidate genes was assessed by RT-qPCR across nine centaury developmental stages, including embryogenic tissues. Notably, a newly reported transcript, named *CeNA1*, was specifically activated during embryogenic callus (**ec**) induction, making it a potential novel marker for early SE. These findings provide, for the first time, insight into SE-related transcriptional patterns, representing a step closer to uncovering the molecular basis of centaury’s developmental plasticity.

## 1. Introduction

Somatic embryogenesis (SE) is the process in which somatic cells are induced to follow an embryogenic developmental pathway, eventually forming somatic embryo (**se**) and complete plant [1,2,3]. The key external factors that induce SE in vitro are various plant growth regulators (PGRs) and/or stress conditions [4]. Most SE induction protocols rely on the use of auxins alone or in combination with cytokinins, with the synthetic auxin 2,4-dichlorophenoxyacetic acid (2,4-D) proving to be the most effective and, therefore, most commonly used [5]. SE can occur directly on the explant, or indirectly, with an embryogenic callus (**ec**) interphase [6].

At a molecular level, SE involves significant transcriptome modifications, epigenetic changes, and frequent re-entries into the cell cycle, which is particularly evident in the early stages of indirect SE [6]. The genes involved in auxin indole-3-acetic acid (IAA) synthesis via the tryptophan pathway, such as tryptophan aminotransferases (*TAA1*/*TAR*) and YUC flavin-dependent monooxygenases (*YUCCA* (*YUC*)), as well as conjugation components like acyl acid amido synthetases *GRETCHEN HAGEN 3* (*GH3*) [7] and signaling components like the *Aux/IAA* and *ARF* families, are often activated (reviewed in [5]). Additionally, the activity of most transcription factor (TF) coding genes is linked to responses to PGRs, highlighting their crucial role in regulating SE [8]. Several TFs typically involved in seed development can induce SE, such as *LEAFY COTYLEDON* (*LEC1, LEC2*), *FUSCA 3* (*FUS3*) [9,10], *AGAMOUS-LIKE 15* (*AGL15*) [6], *WUSCHEL* (*WUS*) [11], and *BABY BOOM* (*BBM*) [12]. The signaling component *SOMATIC EMBRYOGENESIS RECEPTOR KINASE 1* (*SERK1*) is the most well-known marker of SE initiation [4]. Despite these insights, the precise mechanisms underlying this remain partially understood, particularly regarding how these factors interact at different developmental stages and how they are regulated. Recent findings on transcriptional regulation in *Arabidopsis thaliana* revealed that many genes involved in SE, including master regulators, auxin, and stress-related genes, are regulated by histone acetylation, providing insights into the complex epigenetic network that controls embryogenic transition [13]. Besides protein-coding genes, certain classes of non-coding RNAs can also affect SE induction; for instance, it was shown that the microRNAs miR165/166 and miR-160 can contribute to auxin-related SE induction [14,15]. Elucidating the genetic basis of SE through a comprehensive approach, including transcriptomics, proteomics, metabolomics, and epigenetic mechanisms, can help to address practical challenges, such as variability in the response to stress and PGRs across species and explant types, embryo growth arrest, and the occurrence of malformed embryos, which further complicate the standardization of SE techniques [16].

*C. erythraea* Rafn., also known as common, European, or small centaury, is a biennial rosette-forming herb from the Gentianaceae family that inhabits most of the northern hemisphere and is commonly found at the edges of forests, as well as on dry and sandy limestone soil [17,18]. Although traditionally known for its numerous medicinal properties (reviewed in [19]), centaury is increasingly becoming a valuable model organism in plant developmental biology research due to its remarkable regenerative potential under in vitro conditions [18]. This characteristic has enabled the development of numerous regeneration protocols, such as SE, micropropagation, organogenesis, cell suspension, shoot, and hairy root cultures (reviewed in [18]). The most common method of centaury regeneration in vitro is SE, which can occur through various pathways influenced by factors such as light conditions, the types and concentrations of PGRs, and the plant tissues used as explants. Barešová and Kaminek first induced SE in centaury from a callus-derived cell suspension under light, using kinetin and 2,4-D or IAA [20]. Subsequently, primary SE was established from root and leaf explants on Murashige and Skoog (MS) medium or with specific PGR combinations under light/dark regimes [21,22,23,24]. In addition to primary SE, secondary and cyclic SE were established on leaf explants cultivated in darkness using different combinations of 2,4-D and CPPU, with the combination of 0.1 mgL^−1^ 2,4-D and 0.25 mgL^−1^ CPPU proving to be the most successful for embryo progression through cycles, according to Bogdanović et al. [25].

Although centaury has been extensively studied for its phytochemical composition and regenerative potential, the genetic mechanisms underlying its SE remain unexplored. This knowledge gap exists primarily due to the limited genomic resources and the polyploid nature of centaury [26,27]. Elucidating the molecular basis of SE would advance centaury as a model for plant developmental biology and enhance its biotechnological applications, particularly in optimizing in vitro regeneration techniques for the mass production of bioactive compounds and genetic transformation protocols. In addition, efficient in vitro techniques are important for sustaining this species, as it produces small, positively photoblastic seeds [28], which can make it challenging for it to thrive in dense populations. Moreover, this will contribute to a broader understanding of the SE mechanisms across diverse plant taxa, offering insights into various evolutionary and developmental processes.

By analyzing the differential gene expression profiles across distinct SE developmental stages, this research aims to uncover novel molecular markers and elucidate the genetic basis of SE using recently published next-generation RNA sequencing (RNA-seq) data from our team [23]. We address research questions about the genes and pathways differentially expressed during centaury SE, their relation to known mechanisms of embryogenic competence and development in plants, and the potential of novel transcripts from RNA-seq data as reliable SE markers.

## 2. Results

### 2.1. In Silico Identification of DEGs During SE and Organogenesis in Centaury Transcriptome

**Figure 1:**
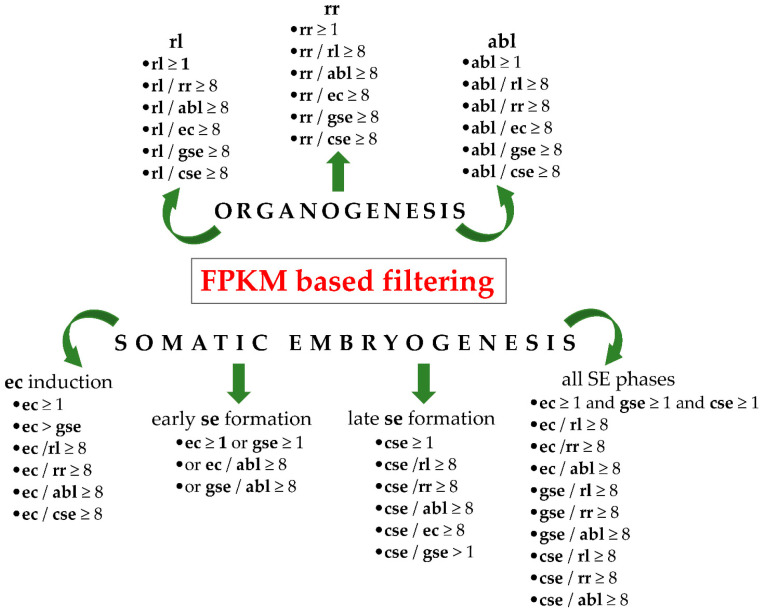
Identification of differentially expressed genes (DEGs) during organogenesis and somatic embryogenesis (SE) of centaury within transcriptome data. **rl**—rosette leaf, **rr**—rosette root, **abl**—adventitious bud, **ec**—embryogenic callus, **gse**—globular somatic embryo, **cse**—cotyledonary somatic embryo, and FPKM—fragments per kilobase of transcript per million mapped reads. Transcriptome was published by Ćuković et al., 2020. [23].

The identification of genes with differential expression provides insight into the specialized developmental processes in target tissues. With this aim, a total of 160,839 pooled centaury transcripts from in vitro induced organogenic tissues, rosette leaf (**rl**), rosette-root (**rr**), and adventitious bud (**abl**), as well as from embryogenic tissues, embryogenic callus (**ec**), globular somatic embryo (**gse**), and cotyledonary somatic embryo (**cse**), underwent careful filtering to identify the DEGs during organogenesis and SE. The filtering criteria (Figure 1) were based on differences in the expression levels among tissues, represented as fragments per kilobase of transcript per million mapped reads (FPKM).

**Figure 2:**
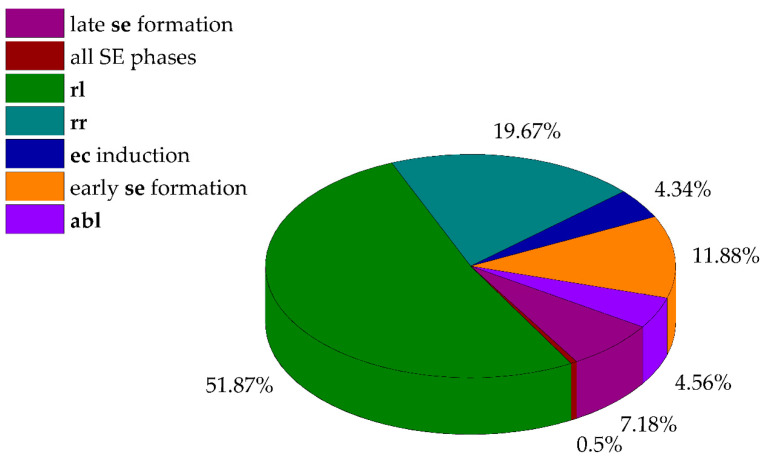
Distribution of tissue-specific DEGs involved in organogenesis and somatic embryogenesis in centaury transcriptome. The percentages were calculated in relation to the total number of DEGs (16,748).

Filtering led to the identification of seven gene subsets (Figure 2, data deposited at the institutional data repository, https://hdl.handle.net/21.15107/rcub_ibiss_7120), with total of 16,748 hits. The majority of DEGs were identified in **rl** and **rr** tissue samples; of these, more than half of the identified genes (8687 or 51.87%) were found in leaves, while 3294 genes (19.67%) were present in roots. A total of 727 genes (4.56%) were detected as being specifically expressed in **abl** samples. Regarding embryogenic tissues, the largest proportion of hits (of 4040 in total) belonged to DEGs detected during the early stage of **se** formation (1989 or 11.88%), while 764 (4.56%) were identified as being specific to **ec** induction. The late stage of **se** formation or DEGs in **cse** samples accounted for 1203 or 7.18% of the total gene number. Finally, only 84 genes (0.5%) were identified in the group of all SE stages, under the strictest filtering conditions (Figure 1).

### 2.2. Functional Annotation of DEGs and Gene Ontology (GO) Enrichment Analysis

As the focus of our research was the identification of specifically active genes during SE, groups of the filtered DEGs in the embryogenic tissues were further examined. The average length of nucleotide sequences was generally similar across groups, approximately 300 bp, except in the library covering all SE stages, where the average length was slightly higher at 507.19 bp (Table 1). When analyzing the annotation data, it is noticeable that the ratio of annotated and unannotated genes, labeled as NA (not available), was somewhat balanced in the NCBI nucleotide (nt) database. However, a higher percentage of unannotated sequences was observed in the Swiss-Prot database, ranging from 60.71% (all SE stages) to 74.98% (late **se** formation).

**Figure 3:**
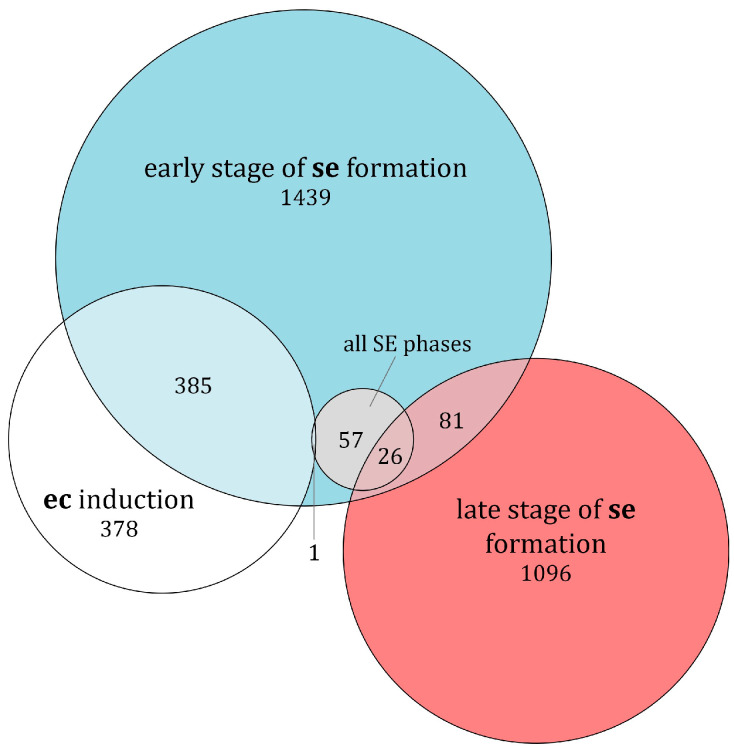
Number of unique and overlapping DEGs during specific SE stages in centaury across four gene subsets.

A substantial number of these genes were present in more than one developmental process (Figure 3). The highest number of genes unique to a single developmental process was found in the early stage of **se** formation (1439), followed by the late stage of **se** formation (1096) and **ec** induction with 378 hits. All genes from the smallest group of all SE stages were subsets of other groups and did not have any unique genes (Figure 3).

**Figure 4:**
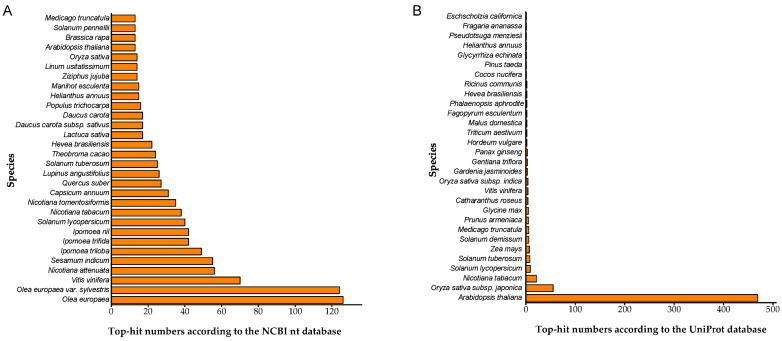
Number of centaury SE-associated transcripts matching 30 top species according to the NCBI nucleotide (nt) database (**A**) and UniProt protein database (**B**).

Since *Centaurium* taxa and other closely related species/taxa were very scarcely present in the nucleotide and protein databases, the majority of annotated SE-related transcripts according to the NCBI nt database showed the highest sequence identity to the *Olea europaea* species and its *sylvestris* variety, with 126 and 124 transcripts, respectively (Figure 4A). Additionally, 70 and 56 transcripts were mapped to *Vitis vinifera* and *Nicotiana attenuata*, respectively. Regarding annotation via the UniProt protein database, the vast majority of transcripts (469 in total) showed the highest similarity to *A. thaliana* sequences. A total of 55 and 21 transcripts were annotated as being similar to *Oryza sativa subsp. japonica* and *Nicotiana tabacum*, respectively, while annotations to other species had fewer than 10 hits (Figure 4B).

**Figure 5:**
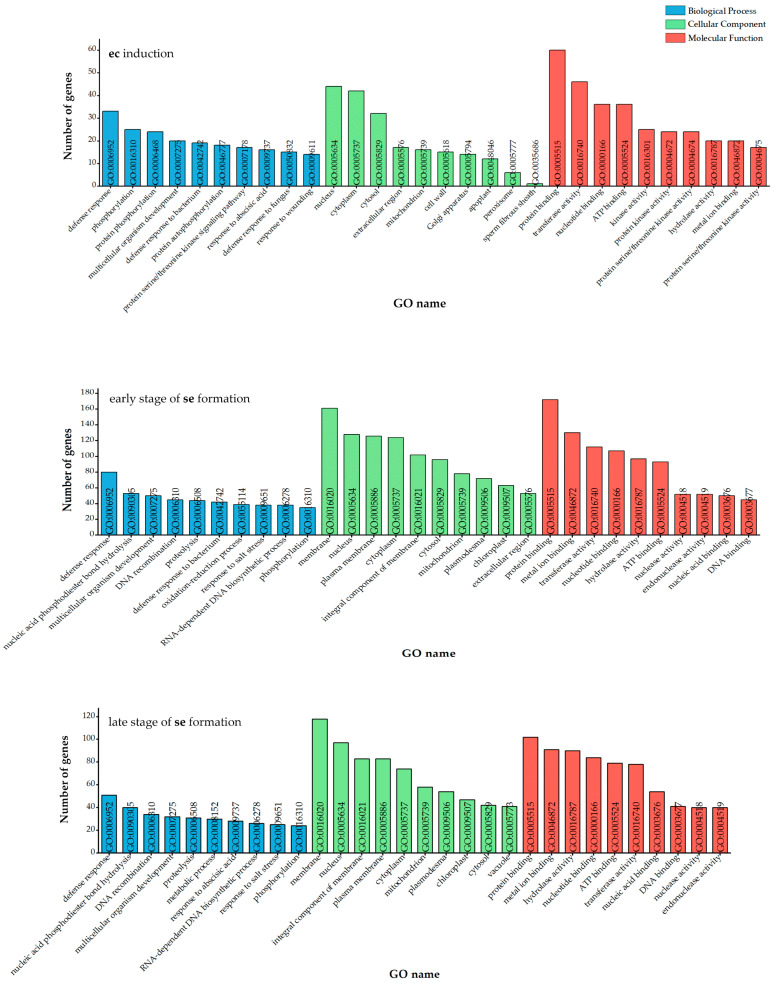
Ten most represented unique Gene Ontology (GO) terms for each phase of SE. All three aspects of GO classification are presented—biological process (blue), cellular component (green), and molecular function (red).

Based on the Gene Ontology (GO) classification, the 10 most frequent GO terms in three categories, biological process, cellular component, and molecular function, were identified for unique DEGs across three SE stages (Figure 5). The dominant biological processes during **ec** induction were associated with the plant defense response. Besides the general defense response term (GO:0006952), more specific terms were also identified, such as response to bacterium (GO:0042742), fungus (GO:0050832), and wounding (GO:0009611). Additionally, a significant number of transcripts were linked to protein phosphorylation (GO:0016310 and GO:0006468) or autophosphorylation (GO:0046777). Similar to the earliest phase of SE, “defense response” was the most represented process in the other two SE phases, followed by the “nucleic acid phosphodiester bond hydrolysis” (GO:0090305) and “DNA recombination” (GO:0006310) in both groups. Additionally, the “multicellular organism development” term (GO:0007275) was represented in all groups (Figure 5). The most prevalent cellular component during **ec** induction was the “nucleus” (GO:0005634), while other significant terms included “cytoplasm” (GO:0005737) and “cytosol” (GO:0005829) (Figure 5). During the early and late stages of **se** formation, the most represented term was “membrane” (GO:0016020), followed by “nucleus” and “plasma membrane” (GO:0005886). The most common term describing molecular function in all groups was “protein binding” (GO:0005515) (Figure 5). The second most represented GO term during **ec** induction was “transferase activity” (GO:0016740), while in other groups, it was “metal ion binding” (GO:0046872). Other significant terms included “ATP binding” (GO:0005524) and “nucleotide binding” (GO:0000166) during **ec** induction, “transferase activity” (GO:0016740) during the early stage of **se** formation, and “hydrolase activity” (GO:0016787) during the late stage of **se** formation (Figure 5).

### 2.3. Selection of Potential SE Marker Genes

**Figure 6:**
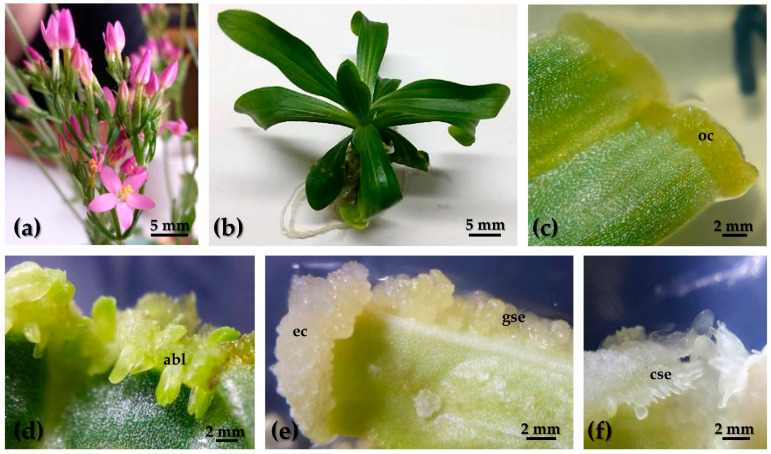
Tissues and organs of *C. erythraea* Rafn. in which the expression of selected gene candidates was evaluated. (**a**) Flowering plants from nature; (**b**) three-month-old plant cultured on MS medium without PGRs; (**c**) leaf explant with **oc** developed on MS medium supplemented with 0.2 mgL^−1^ 2,4-D and 0.5 mgL^−1^ CPPU in light; (**d**) leaf explant with **abl** developed on MS medium supplemented with 0.2 mgL^−1^ 2,4-D and 0.5 mgL^−1^ CPPU in light; (**e**) leaf explant cultured in darkness with **ec** and **gse** developed on MS medium supplemented with 2,4-D and 0.5 mgL^−1^ CPPU; and (**f**) leaf explant with **cse** developed od MS medium supplemented with 2,4-D and 0.5 mgL^−1^ CPPU in darkness. **oc**—organogenic callus, **abl**—adventitious bud, **ec**—embryogenic callus, **gse**—globular somatic embryo, and **cse**—cotyledonary somatic embryo.

In efforts to identify potential SE marker genes in the centaury, the DEGs found in silico were carefully analyzed. With a focus on the FPKM values in the embryogenic tissues and a detailed review of transcriptome annotations according to the Swiss-Prot (part of the UniProt database) and NCBI nt databases, 15 candidates were selected (Table 2) for expression verification on an extended set of tissue and organ samples (Figure 6, Table 3).

**Figure 7:**
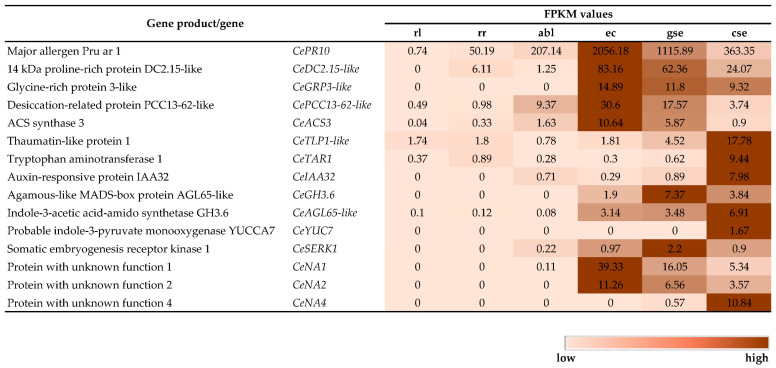
FPKM values of potential SE marker genes in six sequenced tissues of *C. erythraea* Rafn. The color intensity from light beige to dark brown indicates the FPKM expression values for each gene.

**Figure 8:**
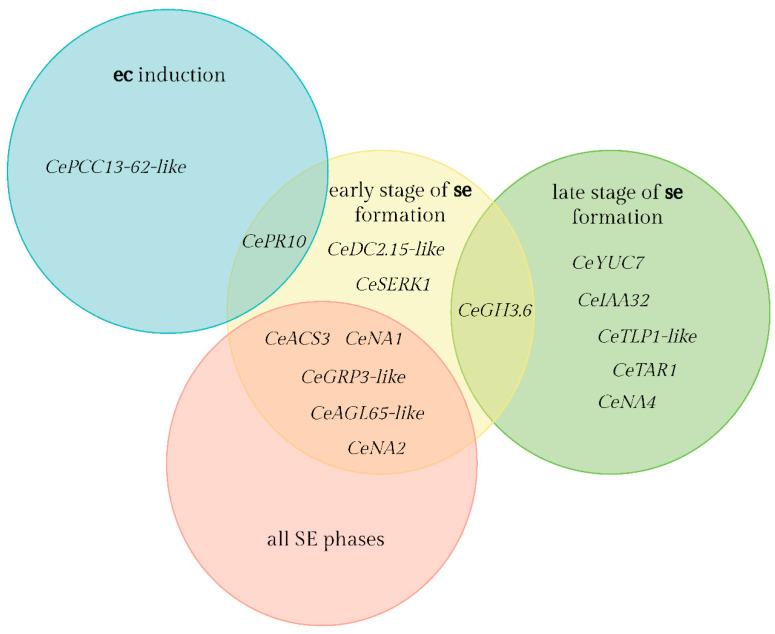
Phases of SE in which selected centaury genes show differential expression based on differences in FPKM expression values.

Some of the identified genes were predicted to be involved in the tryptophan pathway of auxin synthesis, known as the TAA/YUC route. The gene coding for the enzyme TAR1 (*CeTAR1*, TR20674|c0_g3_i1, Table 2), which converts tryptophan into indole-3-pyruvic acid (IPA), had the highest FPKM value in **cse** (9.44), while it’s predicted expression was low in other tissues (Figure 7 and Figure 8). Another gene identified in this pathway was *CeYUC7* (TR46165|c0_g1_i1), with a high protein and nucleotide homology to the *YUC7* gene from *Ipomoea nil* (Table 2). *CeYUC7* was presumably differentially expressed in **cse**, albeit with a low FPKM value of 1.67 (Figure 7 and Figure 8). One of the genes from the *GH3* family involved in auxin metabolism, *CeGH3*.6 (TR39668_c4_g1_i1), was identified in the centaury transcriptome with a high protein and nucleotide homology to the *GH3.6* gene from *Ricinus communis* (Table 2) and the highest FPKM in **gse** (Figure 7). A gene from the *AUX/IAA* family (*CeIAA32*, TR21282|c0_g1_i1), important for auxin signaling, was also selected (Table 2), with a predicted differential expression in **cse** (Figure 7 and Figure 8). Another selected phytohormone-related gene, putatively coding for the enzyme ACS3 involved in ethylene synthesis, was *CeACS3* (TR36872|c0_g1_i2), with the highest FPKM in **ec** (Figure 7) and an E value of zero, indicating an extremely high similarity with the referenced gene (Table 2).

Under the given filtering conditions, the known SE marker gene, *CeSERK1* (TR38192|c1_g2_i6), was found in the centaury transcriptome with a high protein and nucleotide similarity to the *SERK1* gene from *Rosa chinensis* (Table 2). Although with low FPKM values, the presumed expression of *CeSERK1* was ten times higher in **gse** compared to **abl** samples (Figure 7). In addition to the genes necessary for SE initiation, certain genes are involved in **se** development, such as the TF coding gene with a MADS box domain, *AGL*. The *CeAGL65-like* (TR7267|c0_g1_i3) found in the DEG library showed a high protein and nucleotide similarity to the *AGL65* gene from *Nicotiana tomentosiformis* (Table 2). This gene was presumably specifically expressed in **cse**, with an FPKM value of 6.91 (Figure 7 and Figure 8).

According to the centaury transcriptional data, some of the SE-related genes were involved in the stress response, such as genes coding for pathogenesis-related proteins (PRs). Two *PR* genes were found—*CePR10* (TR28332|c0_g4_i1), coding for a protein from the PR10 allergen family, and *CeTLP1-like* (TR30219|c0_g1_i1), coding for thaumatin-like protein 1 (TLP1) from the PR5 family (Table 2). *CePR10* showed the highest recorded FPKM value in **ec** among all the selected candidates (FPKM = 2056.18, Figure 7 and Figure 8) and low Swiss-Prot and nt E values, indicating a high level of similarity with the referenced gene (Table 2). Conversely, *CeTLP1-like* was predominantly expressed in **cse** (Figure 8) with an FPKM value of 17.78 (Figure 7) and a high protein and nucleotide homology to the *TLP1* gene from *O. europaea* (Table 2). Another selected candidate coded for a protein similar to the PCC13-62, *CePCC13-62-like* (TR30412|c0_g1_i1), which was differentially expressed in **ec** tissue samples (FPKM = 30.60, Figure 7 and Figure 8). Based on the low E values from the Swiss-Prot and nt databases, it is presumed that this gene is involved in the desiccation process (Table 2).

Genes coding for a proline-rich protein similar to DC2.15 (*CeDC2.15-like*, TR12274|c0_g1_i1) and a glycine-rich protein (GRP) similar to the GRP3 protein (*CeGRP3-like*, TR3877|c0_g1_i1) were selected as DEGs during the early stages of **se** formation (Table 2, Figure 8). *CeGRP3-like* was presumably specifically expressed in all embryogenic tissues, while its expression was not detected in organogenic tissues (FPKM = 0), whereas *CeDC2.15-like* was activated in the early stages of embryogenesis, with the maximum expression in **ec** (FPKM = 83.16, Figure 7).

For the newly described transcripts named *CeNA1* (TR23240|c0_g1_i1), *CeNA2* (TR48752|c0_g1_i1), and *CeNA4* (TR1001|c0_g1_i1), there were no available annotations in the transcriptome, except for information on transcript length (Table 2). *CeNA1* and *CeNA2* showed a similar trend in their predicted expression, with *CeNA1* having slightly higher FPKM values (Figure 7). Both transcripts were almost undetectable in organogenic tissues, while the highest recorded expression in **ec** gradually decreased through **gse** and **cse**. Conversely, *CeNA4* was presumably differentially expressed in **cse** (FPKM = 10.84), with FPKM values almost undetectable in other tissues (Figure 7 and Figure 8).

### 2.4. RT-qPCR Confirmation of Expression Levels of Selected Candidate Genes

**Figure 9:**
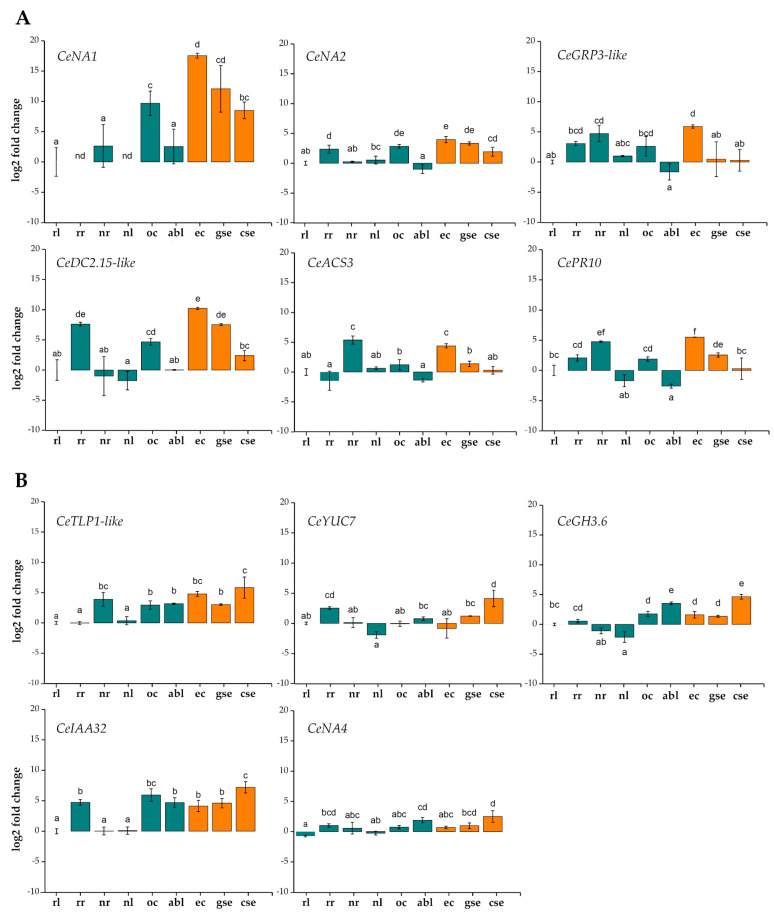
Expression profiles of selected genes in a collection of nine centaury tissues with a potential role in SE. (**A**) Genes active during the induction of embryogenic callus (**ec**) and early formation of somatic embryo (**se**) and (**B**) genes active during late stage of SE, specifically in the cotyledonary-stage embryo (**cse**). In vitro grown rosette leaf tissue (**rl**) was used as the control sample. The mean values ± SE are shown for three biological replicates, and different letters on the graph indicate statistically significant differences compared to the control (*p* < 0.05). Green bars on the graphs indicate samples of different tissues and organs obtained in vitro and from nature, while orange bars indicate tissues of different SE stages. Gene and tissue sample abbreviation explanations are given in Table 2 and Table 3.

**Figure 10:**
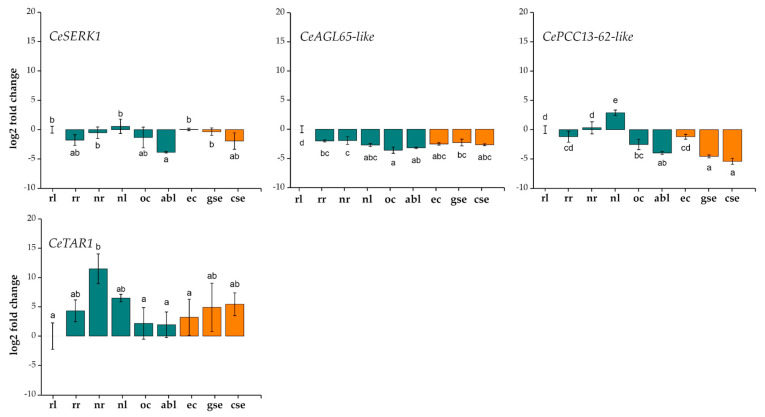
Expression profiles of selected genes in a collection of nine centaury tissues with downregulated gene expression during SE or with expression not significantly altered in most tissues. In vitro grown rosette leaf tissue (**rl**) was used as the control sample. The mean values ± SE are shown for three biological replicates, and different letters on the graph indicate statistically significant differences compared to the control (*p* < 0.05). Green bars on the graphs indicate samples of different tissues and organs obtained in vitro and from nature, while orange bars indicate tissues of different SE stages. Gene and tissue sample abbreviation explanations are given in Table 2 and Table 3.

To experimentally confirm that the genes selected based on the RNA-seq data were indeed differentially expressed in centaury tissues in vivo (Figure 7), RT-qPCR was performed. The results are presented in Figure 9 and Figure 10 as relative changes in gene expression compared to a control rosette leaf sample (**rl**). It was confirmed that some genes participated in the early stages of SE in centaury, such as the induction of **ec** and the early stage of embryo formation, specifically the globular stage, **gse** (Figure 9A). Additionally, some genes were predominantly active in the later stages of embryogenic development, with peak expression in the cotyledonary-stage embryo, **cse** (Figure 9B). Furthermore, a group of genes showed decreased expression during SE, as well as expression that did not significantly change in most samples (Figure 10). Gene expression generally correlated with the RNA-seq results, with minor discrepancies. For example, the *CePCC13-62-like* gene showed a reduced expression in all embryogenic tissues compared to the control sample, while the *CeGH3.6* gene exhibited expression trends that did not align with FPKM expectations (Figure 7 and Figure 9B). Moreover, the gene expression in **abl** and **rr** samples often did not match the predicted low expression levels based on the RNA-seq estimates (Figure 7, Figure 9 and Figure 10).

The most interesting result was the expression profile of the newly described transcript named *CeNA1*, whose expression in **ec** samples not only far exceeded its expression in all other samples, but was about a million times higher than that in the control (Figure 9A). During embryo development, *CeNA1* gene expression remained high, but gradually decreased during **se** development, following the differentiation process. Besides embryogenic tissues, *CeNA1* was also highly expressed in another type of callus sample, organogenic (**oc**), while in differentiated tissues, its expression was significantly lower, with its levels in the **rr** and leaves of flowering plants (**nl**) dropping to levels undetectable by RT-qPCR (Figure 9A). Another unknown transcript, *CeNA2*, was also differentially expressed in embryogenic tissues compared to the control, with its peak expression in embryogenic calluses (approximately 16-fold higher), followed by a gradual decrease in its expression in embryos (Figure 9A). Both *CeNA1* and *CeNA2* exhibited similar expression intensities in **oc** samples as in **ec** ones.

The expression of the gene encoding a glycine-rich protein, *CeGRP3-like*, peaked during **ec** induction (≈32-fold higher than the control) and then drastically decreased during **gse** and **cse** development, reaching control levels (Figure 9A). Additionally, a high expression of this gene was recorded in the roots of flowering plants (**nr**). The expression of the proline-rich protein, *CeDC2.15-like*, exhibited a high expression in the early SE stages, most prominently during **ec** induction (over 1000-fold higher than in the control), which significantly declined in **cse** (Figure 9A). This gene also showed high activity in **rr** samples, at levels near to **gse** ones. The expression of the ethylene biosynthesis pathway gene, *CeACS3*, was significantly higher in **ec** samples compared to the control and other embryogenic tissues, although this gene was most expressed in **nr** (similar to **ec**, Figure 9A).

The pathogenesis-related genes *CePR10* and *CeTLP1-like* were also subjected to RT-qPCR analysis (Figure 9). In embryogenic samples, *CePR10* was most active during **ec** induction (peak expression) and **gse** development, after which, its expression drastically declined to reach control levels in **cse** samples. On the other hand, the transcription of the *CeTLP1-like* gene was activated in all embryogenic tissues (**ec**, **gse**, and **cse**), **oc,** and **abl** samples, compared to the control, with the maximum expression recorded in **cse** (≈32-fold, Figure 9B). Both genes exhibited notable expressions in **nr** tissues, comparable to **ec** (Figure 9).

The expression of the auxin-related genes *CeYUC7* and *CeIAA32* was highly activated during **cse** development (Figure 9B). Additionally, their expression markedly differed between in vitro conditions and tissues from nature. In all tested organs collected from nature, the expression of these genes was very low—at or below the control level (Figure 9B). Another auxin-related gene, *CeGH3.6*, was most highly expressed in **cse** and **abl**, with a statistically significantly increased expression compared to the control and other tissues (Figure 9B). The expression of the third unknown transcript, *CeNA4*, reached its peak in **cse** samples, which is a statistically significant increased expression compared to the control and other embryogenic tissues (Figure 9B).

Some of the tested genes showed a low expression in all or certain embryogenic tissues, often below the level of the control sample (Figure 10). The expression of the *CeSERK1* gene in centaury was down-regulated in embryogenic tissues, with a noticeable decreasing trend in most other tissues, reaching a minimum in **abl** samples. The gene associated with desiccation, *CePCC13-62-like*, not only exhibited a reduced expression in most tissues compared to the control, but this difference was most pronounced (statistically significant) in **gse** samples, and particularly in **cse** ones. *CePCC13-62-like* was most active in the leaves of flowering plants, **nl**. *CeAGL65-like* showed a reduced expression compared to the control in all tested tissues. In the case of the gene auxin biosynthesis component, *CeTAR1*, a statistically significant increased expression in **nr** tissue samples was noticed (Figure 10).

**Figure 11:**
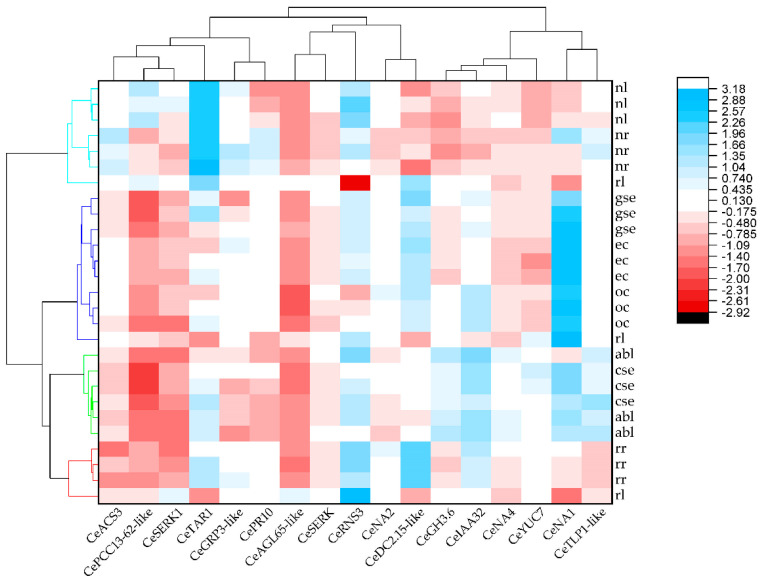
Heatmap showing the expression profiles of 15 SE-related genes in nine tissue and organ samples of centaury. The color spectrum from red to blue represents log2-transformed changes in expression compared to the control **rl** sample; red and blue colors represent decreased and increased levels of expression, respectively. Four main tissue clusters are colored for easier visualization.

A different representation of these expression variations is presented on a heatmap, visually displaying the correlations between the relative expression (log2 fold change) of the tested genes and tissue samples (Figure 11). A leaf sample, **rl**, was used as a control for normalizing gene expression. Genes and tissues were grouped based on similarity, with color intensities illustrating the amplitude of their expression (red—decreased and blue—increased relative to the control). Dendrograms provide additional information on the relationships between samples and genes, offering a comprehensive overview of the expression patterns of the tested genes. Clustering identified four groups of tissues that shared similar expression patterns (Figure 11). One cluster included samples of both callus types (**ec** and **oc**), and **gse**, **cse,** and **abl** samples formed another distinct cluster. The third group consisted of in vitro derived root samples, **rr**. Finally, flowering plants collected from nature exhibited a similar expression pattern among themselves, while differing from that of in vitro cultivated plants. Leaf and root samples from nature (**nl** and **nr**) formed the fourth, separate cluster (Figure 11).

## 3. Discussion

### 3.1. Identification and Characterization of SE-Related Genes in Centaury Transcriptome Data

The ability of *C. erythraea* to undergo SE demonstrates its remarkable developmental plasticity, through its capacity to regenerate through multiple in vitro pathways, including primary, secondary, and cyclic SE [20,21,22,23,25]. This versatility highlights its ability to reprogram somatic cells into embryogenic pathways, which is a feature of developmental plasticity. Its high adaptability is further highlighted by the induction of cyclic SE, where embryos repeatedly progress through successive developmental SE cycles [25]. However, despite success in inducing SE from various explants using combinations of PGRs and research at the protein level, such as the role of arabinogalactan proteins [29,30,31,32], the genetic and epigenetic regulation of SE remains unexplored until now. This could be due to the lack of a sequenced genome and annotations for genetically similar species, as well as the polyploidy of centaury. Some of the approaches for overcoming these challenges include the de novo transcriptome assembly improvement of RNA-seq data quality and coverage, comparative genomics with closely related species from the Gentianaceae family, which could help to annotate uncharacterized genes, as well as the functional validation of candidate genes via techniques like gene expression knockdown, genome editing, or overexpression studies. Overcoming this issue is important not only for a better understanding of centaury’s plasticity, but also for improving methods for its in vitro propagation and genetic transformation, as well as across other plant species.

NGS technology is a powerful platform now routinely used in non-modal species to detect transcripts in various tissues, thus enabling the analysis of the different expression profiles closely associated with specific biological processes [33]. By comparing expressions in this way, it is possible to gain insights into the gene activity under different conditions, namely, to identify DEGs. To identify the genes that are specifically activated during SE in centaury, we investigated RNA-seq data comprising 160,839 centaury transcripts originating from organogenic (**rl**, **rr,** and **abl**) and embryogenic (**ec**, **gse,** and **cse**) tissues. The identification of DEGs was based on criteria related to differences in transcript FPKM values. In this context, it should be noted that not many studies rely solely on differences in FPKM values for DEG selection. One such example is the identification of tissue-specific genes during rhizome development in *M. lutarioriparius*, where all genes with FPKM > 10 and at least a three times higher expression compared to other tissues were selected [34].

The majority of identified hits (16,748 total) with predicted tissue-specific expression belonged to the **rl** (51.87%) and **rr** (19.67%) tissue samples, which was expected given that they are fully differentiated tissues. Since SE is a highly specialized process, the number of up-regulated genes in embryogenic tissues was significantly smaller, 4040 in total, with some genes not being unique to a specific condition due to the filtering criteria. These results are consistent with other projects that have analyzed the transcriptome during SE, such as a transcriptome analysis of mangosteen (*Garcinia mangostana*) using Trinity technology, where 4001 DEGs were identified during SE out of a total of 186,203 transcripts [35]. The identified DEGs during SE were classified into specific SE stages based on FPKM values, with 378 genes being uniquely present during **ec** induction, 1439 during early **se** development, and 1096 in late **se** development. Chen et al. reported 366, 505, and 588 unique genes in **ec**, incomplete pro-embryogenic cultures, and the globular **se** stage in Longan [36]. Similarly, in upland cotton, 786 unique DEGs were found in **ec** and 545 genes with altered expression were identified in a mixed sample containing various **se** stages [37]. The varying numbers of DEGs during SE in centaury indicate different transcriptional patterns across various developmental processes, which provides insight into the specific biological processes occurring in the individual stages of embryogenic development.

According to the NCBI nt database, centaury genetic material shares some level of homology with *O. europaea*, as the sequences showed the highest similarity to this species. As for the UniProt database, most transcripts were annotated to *A. thaliana*, since it is a commonly used model organism in plant biology, with the most annotations in the UniProt database (https://www.uniprot.org/uniprotkb/statistics). Homologs were found in the NCBI nt database for less than 50% of DEGs in each SE stage, while this number was even smaller in the Swiss-Prot database (≤40%), with only 25.02% of annotated transcripts in **cse** samples, indicating a large number of sequences without obvious homologs. This result suggests that some of these genes are likely novel genes. The challenge of annotation was further hindered by the lack of centaury functional genomic data, which further complicated the experimental validation of predicted gene functions or the discovery of new functions.

In all stages of SE in centaury, the most represented GO terms describing biological processes related to plant defense responses. The link between stress and SE is well documented, with SE in some plant species being routinely induced by stimuli such as wounding, high salt concentrations, heavy metal ions, or osmotic shock [38]. Notably, SE is considered as an adaptive stress process resulting from massive cellular restructuring and the reprogramming of gene expression in treated tissues [38].

### 3.2. Expression Patterns of Gene Candidates in Embryogenic and Vegetative Tissue Collection

The heatmap in our study showed that the majority of biological replicates grouped closely together, with nearly identical expression profiles, which confirms the consistency of the sample collection and the chosen method. These results support the existence of common regulatory networks in centaury that are characteristic for specific tissues or organs. In addition, many of selected candidate genes are multifunctional, i.e., they are activated in accordance with the current physiological status of the plant, where they can regulate various biological processes. Genes active during the induction of embryogenic calluses and the early formation of somatic embryos in centaury primarily showed an elevated expression during **ec** and/or **gse** development, indicating their role during the earliest stages of SE induction and young embryo formation (Figure 9A). Although cells within the callus may exhibit varying degrees of differentiation, a callus is most commonly considered to be a manifestation of a dedifferentiated state in the cell, characterized by a high rate of cell proliferation [39]. The main characteristic of dedifferentiation is the regression from a previously differentiated state to a less specialized form, where cells gain competence and can re-enter the cell cycle. This further determines their developmental path, either towards differentiation into specific cell types or towards commitment to cell death [40,41]. Embryogenic callus cells arise as a result of the intense divisions of somatic cells that are cultured in vitro, typically influenced by PGRs and stress [42]. According to Wójcik et al., 2,4-D was used alone or in combination with other PGRs in more than 78% of protocols for SE induction [5]. In addition to being a plant growth regulator, 2,4-D is also a herbicide at higher concentrations, and these contrasting properties are most likely the reason for its effectiveness in inducing an embryogenic response [5]. The application of 2,4-D efficiently induces in vitro morphogenetic responses through cell division and plant growth, usually at low concentrations of 1–10 mgL^−1^ [5,43]. In this study, centaury embryogenic tissues were successfully induced on leaf explants after four weeks in darkness, in the presence of 0.2 mgL^−1^ 2,4-D together with 0.5 mgL^−1^ CPPU. At the molecular level, 2,4-D induces endogenous auxin biosynthesis via the TAA1/YUC pathway in competent cells, which results in the cell division that ultimately leads to the development of globular structures [44]. In addition to 2,4-D, other synthetic auxins, such as Picloram and Dicamba, can also be used for SE induction [45].

The expression profile of the previously unreported transcript *CeNA1* suggests that this gene could be a newly discovered marker of SE in centaury, particularly in the earliest stage (Figure 9A). It is essential that any gene marker used to identify the earliest stage of SE is reliable, sensitive, and shows a high expression specifically at that stage [46,47]. *CeNA1* was specifically active in all embryogenic tissues, with its expression gradually decreasing during **se** development, following the differentiation process. The exceptionally high level of *CeNA1* expression in **ec** samples indicates that its activity was characteristic for the transition from a somatic to embryogenic state. Its high expression in undifferentiated callus tissues, which decreased in embryos, as well as its low or undetectable expression in other samples, suggests that *CeNA1* gene activity could positively correlate with the intense cell proliferation occurring during callus induction, especially in **ec**. Its discovery is important, as it could potentially offer new perspectives for improving the efficiency and success of in vitro regeneration. The ectopic expression of SE-related genes could enhance the embryogenic potential of somatic cells through constitutive or transient expression, enabling the propagation of elite lines reluctant to regenerate and produce transgenic plants [48]. Also, molecular markers can be used for improving plant breeding through the more effective selection of plants with desirable traits [4]. However, although there are indications that *CeNA1* could be a newly discovered marker of SE, it is necessary to validate its function in the future through functional assays such as expression knockdown, overexpression, or genome editing to confirm its reliability.

Another unknown transcript, *CeNA2*, showed a similar expression pattern to *CeNA1* in embryogenic tissues and **oc** samples, but with a reduced intensity and an extremely low expression in other samples, suggesting its potential activity during the early stages of SE. On the contrary, the expression of *CeNA4* peaked in **cse** samples (Figure 9B), which suggests a potential role for this gene in the process of differentiation and embryo formation, or possibly in the maturation of **cse**. As with *CeNA2*, these preliminary assumptions about *CeNA4*’s possible function should be further investigated.

Since the initially described plant GRPs were located in the cell wall [49,50], these proteins are mainly associated with structural functions. However, due to their great diversity in structure, expression patterns, and intracellular localization, they could be involved in various processes in plants, including SE [51,52]. The first isolated *GRP* gene, *CEM6*, from *Daucus carota*, which encodes a cell wall protein, was differentially expressed during the early stages of SE, predominantly from the globular to the heart stage, with its expression gradually decreasing during **se** development [53]. High activity of the *GRP5* gene, which encodes a protein similar to CEM6, has been observed in all cells of the proembryogenic mass and **se** up to the torpedo stage, while during the transition to the cotyledonary stage, this expression gradually decreased [54]. The expression of *CeGRP3-like* was highly activated in **ec** samples, and then notably excluded at the beginning of **se** development, suggesting that this gene is active in cells undergoing dedifferentiation before embryo formation begins. The significant expression of *CeGRP3-like* in roots from nature, **nr**, indicates a probable role of this gene in pathogen defense. Namely, some of the identified *GRP* genes are expressed in roots and have antimicrobial activity, such as *CbGRS1* from *Capsella bursa-pastoris* [55]. Unlike the *CeGRP3-like* gene, whose expression was limited to **ec** samples, the *CeDC2.15-like* gene was active in centaury **ec** cells and young embryos undergoing the first anatomical modifications leading to the formation of mature **se**. DC2.15 proteins primarily function in linking the cell wall and membrane, contributing to adhesion, signaling, and maintaining cell structure [56]. They were first identified during SE in a carrot cell suspension, where *DC2.15* expression was initially linked to the early morphogenetic events in SE, especially during the globular and heart stages [57]. Consequently, a high expression of *CeDC2.15-like* during early SE may be associated with intense centaury cell division. The differential expression of the *CeDC2.15-like* gene in **rr** tissues also suggests its possible specificity and regulation at different developmental stages, which may indicate a potential role in responding to the specific physiological demands of plants under various conditions.

Results from numerous studies suggest that ethylene biosynthesis can influence both SE induction and **se** formation, although the molecular mechanisms underlying these processes are not yet fully understood (reviewed in [58]). Specifically, the effect of ethylene on SE is often contrasting, depending on the species and even the SE stage. For instance, in alfalfa, ethylene promotes both **ec** proliferation and **se** development [59,60], while in spinach, it induces **ec** induction, but inhibits **se** development [61]. In centaury, an increased expression of *CeACS3*, necessary for ethylene synthesis, was observed in **ec** samples, with a significant decrease during **se** development, eventually reaching control levels in **cse**. The induced expression of *CeACS3* seems to be associated with the application of 2,4-D, as initial stress stimuli caused by auxins, such as 2,4-D, are known to increase *ACS* expression and enhance ethylene synthesis [62,63]. According to Bai et al. [64], higher initial levels of ethylene induced by auxins appear to suppress the genes involved in the auxin biosynthesis pathway, leading to a reduced number of developed **se** in *Arabidopsis*. Besides its high expression in **ec** samples, a high expression of *CeACS3* was also noted in roots from nature, **nr**. Previous studies on Sitka spruce roots have shown that insect attacks or mechanical injuries cause the strong induction of *ACS3* and *ACS2* genes [65], suggesting that this high *CeACS3* expression in **nr** may have been the result of a similar defense mechanism in centaury.

PR proteins are part of the plant defense responses encoded by genes that are rapidly induced in response to biotic and abiotic stress and the accumulation of phytohormones associated with defense mechanisms [66]. Although most PR proteins exhibit antimicrobial activity, many studies have indicated their possible additional role in plant development, including SE [67,68,69,70]. PR10 proteins have been shown to be the most abundant proteins induced by 2,4-D treatment, leading to SE in *M. truncatula* [68,71]. Also, two transcripts similar to *TLPs* from the earliest SE stages in carrot preferentially accumulated in embryogenic clusters [72]. Both tested centaury *PR* genes were expressed during SE, with spatially different transcription patterns. While *CePR10* was active up to the globular embryo stage, after which it was turned off, *CeTLP1-like* was expressed at all stages of SE, with a maximum in **cse**. Also, both genes exhibited a high expression in roots collected from nature, **nr**. *CeTLP1-like* expression in tissues cultured in vitro was evidently induced by the presence of 2,4-D. The induction of certain defense reactions is not surprising, given that 2,4-D is known to cause oxidative stress in treated tissues [73]. The elevated expressions of both genes in **nr** tissues were most likely due to the induction of a basic defense response activated due to the presence of soil pathogens or some type of abiotic stress. Numerous studies have confirmed the increased activity of *PR10* and *TLP* genes in response to pathogen attacks, abiotic stress, or fungal infections, such as the *PR10* genes in rubber tree responding to *Rigidoporus microporus* [74] or in strawberry plants responding to *Verticillium dahliae* [75].

Several genes important for auxin biosynthesis, metabolism, and signaling showed significant activity during the late embryo formation stage, specifically in the cotyledonary stage, **cse** (Figure 9B). The accumulation of endogenous IAA, together with the exogenous application of auxin, is a crucial factor not only for the induction of SE, but also for **se** development. Increased concentrations of endogenous IAA lead to the re-establishment of gradients and the polar transport of auxin in unorganized callus tissue, which results in the establishment of bilateral symmetry in somatic embryos during the transition from the globular to the heart stage [76]. The formation of the apical shoot and root meristems is accompanied by the organization of cells into embryo-like structures [77]. The TAA/YUC pathway is considered to be the most important pathway for endogenous IAA biosynthesis, where the enzyme TAA1/TAR1 converts tryptophan into IPA, which is then converted into IAA by YUC proteins [78]. In *C. erythraea*, the expressions of two genes from this pathway, *CeTAR1* and *CeYUC7*, were tested. *CeTAR1* did not show specific expression in embryogenic tissue samples, nor significant changes in expression in most other tissues and organs, with significant variations among biological replicates. The potential induction of *YUC7* in SE was first noticed when Saptari et al., through in silico analysis, recorded an increase in its expression from day 5 to day 15 of SE in *A. thaliana* [79]. The expression of the *CeYUC7* gene in centaury was very low in the early SE stages (**ec** and **gse**), but was strongly activated in mature **cse**. Lee et al. demonstrated that the activity of the *YUC7* gene increased drought tolerance in *A. thaliana* and functioned in an ABA-dependent manner [80]. These observations suggest that an increased expression of *CeYUC7* in **cse** may indicate a potential association with the adaptive response to stress conditions, including desiccation, which embryos undergo during the final maturation stage. Members of the *GH3* protein family are involved in the regulation of auxin metabolism by synthesizing IAA conjugates with various acyl groups, which is one of the main mechanisms for maintaining auxin homeostasis [81]. The expression of the *CeGH3.6* gene in centaury embryogenic tissues was most intense during the **cse** stage, and a similar expression intensity was observed in **abl** samples. While *GH3.6* is rapidly and strongly activated in the presence of natural auxins such as IAA or NAA, synthetic auxins like 2,4-D and dicamba have not been shown to induce its transcription [82,83]. It is possible that, due to the constant presence of 2,4-D in our in vitro experiments, the plant does not have an increased need for endogenous auxins, and, therefore, binds them into conjugates, for which the expression of *CeGH3.6* is necessary. By regulating ARF activity, *Aux/IAA* genes are critical components in auxin signal transduction pathways, controlling the precise gene expression necessary for proper plant growth, development, and adaptation to external stimuli [84]. *CeIAA32* was active in **ec**, with a slight but statistically significant increase in expression during the transition to **cse**. Differential expressions of genes from this group (*IAA16*, *IAA29*, *IAA30*, and *IAA31*) have been observed during SE induction in *A. thaliana*, and consistent with these results, mutations in *IAA30* and *IAA31* led to significant defects in the embryogenic response in in vitro cultured tissues [85]. It is presumed that Aux/IAA-ARF mediated responses to auxin also play a role in embryogenic cultures of other plants, including cotton [86,87], rice [88], and cyclamen [89]. The expression of the *CeIAA32* gene during all stages of SE in centaury highlights the importance of auxin signaling in regulating SE induction and development. Additionally, the *CeIAA32* expression profile indicates that it is more active where free auxin is presumed to be higher, such as in the **rr** tissue samples, as well as in tissues affected by exogenous auxins, such as embryogenic tissues and **oc** samples. This observation is further confirmed by the distinctly low expression at control levels in the centaury samples collected from nature, likely due to the fact that they were not exposed to exogenous 2,4-D. Research has shown that the expression of *Aux/IAA* genes is often rapidly induced in the presence of exogenous auxin [90,91], with these genes differing in the sensitivity and nature of their response to auxin [92,93]. Moreover, the expression of specific *Aux/IAA* gene groups can vary significantly between different plant species and even within different tissues of the same plant [94,95]. These observations are consistent with the expression profile of *CeIAA32*, whose expression was not limited to the SE process, but was also active in other tissues with a presumed higher auxin content.

In addition to the genes active during certain stages of SE, the genes *CeSERK1, CeAGL65-like*, and *CePCC13-62-like* showed a low expression in embryogenic tissue samples, often lower than the control (Figure 10). The ability of somatic cells to reprogram development towards embryogenesis involves acquiring embryogenic competence, often induced by stress or PGR treatment, and typically involves an increased expression of *SERK* genes [96]. Since the initial discovery in carrot by Schmidt et al. [97], *SERKs* have been reported in both the zygotic embryos and **ses** of numerous plant species, including Arabidopsis [98], rice [99], and maize [100], highlighting their role in embryogenesis across diverse plant species [101]. Moreover, it has been shown that the overexpression of *SERK1* enhances the embryogenic response in *A. thaliana* [98]. Therefore, *SERK* is considered to be a marker of embryogenic competence in plants [101]. The expression of the *CeSERK1* gene in centaury was down-regulated in embryogenic tissues, and these values corresponded to the expected FPKM values. The presented results confirmed that transcripts with higher FPKM values should be preferred when choosing markers for a biological process. Transcripts with low FPKM values are likely of lesser biological significance [102]. Some of these transcripts may have had falsely low or zero expression values due to incomplete assembly or a lack of coverage at both read ends, and, therefore, cannot be reliably considered. The obtained results confirmed that the chosen *CeSERK1* gene is not a marker of SE in centaury. However, it is possible that selecting genes with different sequences, e.g., more distant relatives of these genes, could lead to the detection of genes with an increased expression in embryogenic tissues. *CeAGL65-like* is presumed to encode a TF with a MADS domain, which is most commonly involved in regulating various developmental processes in plants, such as regulating flowering time and organogenesis, fruit development, and seed maturation [103]. Additionally, the *AGL* family gene, *AGL15*, is known as one of the central regulators of SE induction, which it achieves through interaction with numerous TFs and the regulation of auxin, ethylene, gibberellin, and brassinosteroid signal transduction [6]. However, the *CeAGL65-like* gene showed an extremely low expression in most tested centaury tissue and organ samples, including embryogenic tissues. The fact that some common genes associated with SE in centaury did not show an altered expression suggests that they may not be involved in this process in the same way as in other plants. This also indicates the possibility that other genes in centaury may be responsible for the regulation and control of this process. Another gene that did not meet FPKM expectations was *CePCC13-62-like*. Desiccation-related proteins are characterized by accumulation during desiccation, which is associated with dehydration tolerance [104]. *CePCC13-62-like* showed the lowest expression in centaury embryos, with its maximum expression in the **nl** tissue samples. *PCC13-62* gene transcripts abundantly accumulate in the dehydrated tissues of plants tolerant to dehydration, such as *C. plantagineum* and *L. brevidens*, while in the dehydrated leaves of plants sensitive to dehydration, like *L. subracemosa*, they are expressed, but to a much lesser extent [104]. Based on these results, it can be proposed that the elevated expression of *CePCC13-62-like* in the **nl** samples likely represented a dehydration response, which can be explained by the lack of selective pressure for desiccation tolerance in the centaury habitat [28,105]. In the same manner, the extremely low expression in embryogenic tissues can be explained by the high sensitivity of these tissues to dehydration.

## 4. Materials and Methods

### 4.1. Transcriptional Analysis and Identification of DEGs

In this study, an RNA-seq dataset previously published by Ćuković et al. [23] was acquired from the Zenodo research sharing platform (data deposited at open digital repository, https://zenodo.org/record/3591805). In brief, the six following tissues were sequenced on the HiSeq 2500 Illumina platform: rosette leaves (**rl**), rosette roots (**rr**), adventitious buds (**abl**), embryogenic calluses (**ec**), globular somatic embryos (**gse**), and cotyledonary somatic embryos (**cse**). High-quality reads from all samples were pooled and used as inputs for de novo transcriptome assembly via Trinity technology. The assembled “raw” centaury transcriptome was 142 Mb in size and contained 160,839 Trinity transcripts. The quality and coverage of the transcriptome were assessed using BUSCO (v3) analysis [106,107], which showed that all but two BUSCO genes were found in the transcriptome, with 94.9% complete BUSCO genes, which indicates that the *C. erythraea* transcriptome was of a very high quality [23]. Gene expression was normalized as FPKM values [108], which were used as the criteria for selecting DEGs. For tissue-specific genes in the **rl**, **rr**, and **abl** samples those with an FPKM value of at least one in the target tissue and an expression level at least eight times higher than that in all other tissues were selected (Figure 1). Four additional filtering criteria were set to detect active genes during SE. For DEGs during **ec** induction, genes with FPKM ≥ 1 in **ec**, a higher expression in **ec** than in **gse**, and an expression level at least eight times higher in **ec** than in **rl**, **rr**, **abl,** and **cse** were selected. For genes potentially active during the early stages of **se** formation, those with an FPKM ≥ 1 in **ec** or **gse** or at least an eight times higher expression in one of them compared to **abl** were selected. DEGs during the late phase of **se** formation were identified as genes with FPKM ≥ 1 in **cse**, a higher expression in **cse** than in **gse**, and at least an eight times higher expression in **cse** compared to **rl**, **rr**, **abl**, and **ec**. Finally, a group of genes meeting the strictest criteria was selected, as follows: FPKM ≥ 1 in **ec**, **gse**, and **cse**, and at least an eight times higher expression in each embryogenic tissue compared to organogenic tissues (**rl**, **rr,** and **abl**) (Figure 1). A Venn graph was drawn using an online tool Visual paradigm, v.17.2. (https://online.visual-paradigm.com/).

Sequences with hits in the NCBI nt and Swiss-Prot databases were further loaded into UniProt [109] and Blast2GO, v6.0 [110] to obtain detailed functional information, including GO classification and PFAM protein domain identification. In the GO terms analysis for each gene group, only unique hits for a specific subset were considered. General terms like “biological process” or “molecular function” were excluded from the graphs due to insufficient informativeness.

When detecting genes with the most appropriate characteristic regarding SE, special attention was given to sequences with functional annotation that had suitable E values and lengths. In this case, only sequences with an E value of < 1 × 10^−15^ according to the Swiss-Prot database were considered, given that a lower E value indicates a more biologically accurate annotation [111]. When the Swiss-Prot and NCBI nt database predictions were contradictory, priority was given to the Swiss-Prot database.

### 4.2. Plant Material

A collection of nine centaury tissues and organs from different developmental stages (Figure 6, Table 3) was prepared according to Ćuković et al. [23] and used for the expression analysis. These samples included in vitro grown rosette leaves (**rl**) and rosette roots (**rr**) from three-month-old plants, adventitious buds (**abl**), organogenic calluses (**ocs**), embryogenic calluses (**ec**), globular embryos (**gse**), and cotyledonary embryos (**cse**), as well as leaves (**nl**) and roots (**nr**) from plants growing in nature. Rosette plants were cultured from *C. erythraea* Rafn. commercial seeds (Jelitto Staudensamen GmbH, Schwarmstedt, Germany) on MS medium [109] without PGRs under light conditions. **oc** and **abl** tissues samples were collected on leaf explants after four weeks on medium supplemented with 0.2 mgL^−1^ 2,4-D and 0.5 mgL^−1^ CPPU in light, while embryogenic tissues, **ec**, **gse,** and **cse**, were induced from leaf explants after four weeks on the same medium, but in darkness (Table 3).

### 4.3. Evaluation of the Expression of SE-Related Genes

Gene sequences with differential expression during SE were found in the centaury transcriptome using local BLAST, with some of these genes present in multiple isoforms. Different isoforms of the same gene were aligned via BioEdit Sequence Alignment Editor software, v. 7.2.6.1 [112]. Primers were designed using Primer Blast (https://www.ncbi.nlm.nih.gov/tools/primer-blast/) on the sequence region of the selected isoform that did not belong to the conserved region, ensuring the specificity of amplification only for the selected isoforms and avoiding unwanted amplifications. The primer quality was checked on the NetPrimer website (https://www.premierbiosoft.com/netprimer/), with special attention paid to the detection of secondary structures, such as hairpins, homodimers, and cross-dimers. Primer specificity was experimentally confirmed by agarose gel electrophoresis of the RT-PCR products. The sequences of primers for the selected DEGs are given in Table S1.

A cDNA collection of the nine tissues and organs was prepared according to the procedure described by Ćuković et al. [23]. Gene expression was evaluated by real-time quantitative RT-qPCR with a Maxima™ SYBR Green/ROX qPCR Master Mix 2x kit (Thermo Fisher Scientific, Waltham, MA, USA). Each reaction had a volume of 10 μL, containing 0.3 µM specific primers and cDNA corresponding to 12.5 ng RNA, and was performed in three biological replicates. For each gene, a negative control was used, with 1 μL H_2_O instead of cDNA. The QuantStudio^®^ 3 Real-Time PCR System (Thermo Fisher Scientific, Waltham, MA, USA) was used for amplification. The amplification parameters included initial denaturation (95 °C/10 min), followed by 40 cycles of denaturation (95 °C/15 s), hybridization (at gene-specific Ta/30 s), and extension (72 °C/30 s). A final extension at 72 °C for 10 min was followed by a melting curve analysis.

The gene expression levels were determined as cycle threshold values (Cts) and further analyzed using relative quantification. Genes coding for ribosomal protein L2 (*RPL2*) and TATA-binding protein 1 (*TBP1*) were used for the normalization of target genes, as they were previously shown to be the most stable reference genes in most developmental processes in centaury, including SE [23]. Standards for the standard curve were made by the serial dilution of purified PCR fragments in the range of 10^9^–10^2^ copies. Based on the standard curve parameters, the RT-qPCR reaction efficiencies were found to be within the optimal range of 90–110%.

### 4.4. Data Collecting and Statistical Analysis

The expression of presumably differentially expressed genes during SE was analyzed using relative expression quantification (log2 scale) by the 2^−ΔΔCt^ method according to [113]. A leaf tissue sample (**rl**) was used for the calibration of gene expression changes in other tissues, and these values were logarithmically transformed and subjected to ANOVA followed by a Tukey post hoc test to identify statistically significant differences (*p* < 0.05). When the expression was out of the detection range, the y-intercept value was taken as the Ct value.

To visualize variations in the expressions of differentially expressed genes, the hierarchical clustering of tissue samples was performed. The clustering method was based on Euclidean distances (a value of 0 indicates complete identity, while higher values indicate greater differences) constructed based on relative expression changes (log2 scale). Ward’s method of cluster agglomeration was used [114]. The similarity between pairs of gene expressions was estimated using Pearson correlations and the Furthest Neighbor cluster method. The correlation table was presented as a heat map using the Origin 2019b tool Heat Map with Dendrogram. The complete linkage method was used for cluster agglomeration.

## 5. Conclusions

This study provides the first insight into the transcriptional profile of *C. erythraea* Rafn. during SE. Even though FPKM values from the transcriptome data provide informative guidelines for selecting DEGs, it is recommended to confirm gene expression using a more sensitive method, such as RT-qPCR. By verifying the expression of genes with desirable characteristics, it was confirmed that SE in centaury involves some genes previously identified as being SE-related in other species, as well as several novel genes. Given the network and multifunctionality of genes associated with various aspects of plant growth and development, through the complex process of identifying marker genes for a highly specialized process such as SE, we identified a previously unannotated gene, *CeNA1.* Its differential expression during SE, particularly in embryogenic calluses, suggests that it could be an early SE marker in centaury. This research highlights numerous future perspectives, particularly concerning *CeNA1*, not only to enhance our understanding of the mechanisms that induce and control SE in *C. erythraea* Rafn., but also for possible biotechnological applications in the conservation and propagation of other related and recalcitrant plant species, as well as in plant breeding technologies.

## Figures and Tables

**Table 1:** Characteristics of DEG subsets during SE in centaury. NA—not available, unannotated genes.

Characteristics of DEGs During SE	ec Induction	Early Stage of se Formation	Late Stage of se Formation	All SE Phases
Number of DEGs (Swiss-Prot/NCBI nt)	764	1989	1203	84
Number of DEGs With Hits in Swiss-Prot Database (Annotated)	198 (25.92%)	522 (26.24%)	301 (25.02%)	33 (39.29%)
Number of DEGs Without Hits in Swiss-Prot Database (NA)	566 (74.08%)	1467 (73.76%)	902 (74.98%)	51 (60.71%)
Number of DEGs With Hits in NCBI nt Database (Annotated)	338 (44.24%)	870 (43.74%)	518 (43.06%)	42 (50%)
Number of DEGs Without Hits in nt NCBI Database (NA)	426 (55.76%)	1119 (52.26%)	685 (59.94%)	42 (50%)
Average Sequence Length (bp)	310.26	331.19	341.18	507.19

**Table 2:** Selected gene candidates with potential involvement in SE in *C. erythraea Rafn*. The table presents names and abbreviations of proteins and genes along with the accession numbers of transcripts, available annotations according to the NCBI nt and Swiss-Prot databases, E values, and the nucleotide sequence length. Transcripts without annotations are marked as NA.

Gene Product/Gene		Trinity Transcript ID	Protein Homology (Swiss-Prot)	E Value Swiss-Prot	Nucleotide Homology (NCBI nt)	E Value NCBI nt	Length (bp)
Major Allergen Pru ar 1	*CePR10*	TR28332|c0_g4_i1	PRU1_PRUARRecName: Full = Major Allergen Pru ar 1; AltName: Allergen = Pru ar 1	1.03 × 10^−46^	*Solanum tuberosum* Major Allergen Pru ar 1-like (LOC102580863), mRNA	5.43 × 10^−57^	818
14 kDa Proline-Rich Protein DC2.15-like	*CeDC2.15-like*	TR12274|c0_g1_i1	NA	NA	*O. europaea* var. Sylvestris 14 kDa Proline-Rich Protein DC2.15-like (LOC111374281), mRNA	3.3 × 10^−15^	251
Glycine-Rich Protein 3-like	*CeGRP3-like*	TR3877|c0_g1_i1	NA	NA	*Nicotiana sylvestris* Glycine-Rich Protein 3-like (LOC104219161), Transcript Variant X2, mRNA	2.37 × 10^−6^	355
Desiccation-Related Protein PCC13-62-like	*CePCC13-62-like*	TR30412|c0_g1_i1	DRPE_CRAPLRecName: Full = Desiccation-Related Protein PCC13-62; Flags: Precursor	1.05 × 10^−92^	*Capsicum annuum* Desiccation-Related Protein PCC13-62-like (LOC107839603), mRNA	4.34 × 10^−98^	1168
ACS Synthase 3	*CeACS3*	TR36872|c0_g1_i2	1A13_SOLLCRecName: Full = 1-aminocyclopropane-1-carboxylate synthase 3; Short = ACC synthase 3; AltName: Full = Le-ACS3; Short = ACS-3; AltName: Full = S-adenosyl-L-methionine methylthioadenosine-lyase 3	0	*N. attenuata* 1-aminocyclopropane-1-carboxylate Synthase 3 (LOC109218840), mRNA	0	1972
Thaumatin-like Protein 1	*CeTLP1-like*	TR30219|c0_g1_i1	TLP1-like_ARATHRecName: Full = Thaumatin-like Protein 1; Short = AtTLP1	1.39 × 10^−107^	*O. europaea* Var. Sylvestris Thaumatin-like Protein 1 (LOC111401272), mRNA	5.41 × 10^−135^	1197
Tryptophan Aminotransferase 1	*CeTAR1*	TR20674|c0_g3_i1	TAA1_KLULARecName: Full = Protein TAA1	3.68 × 10^−23^	*Armillaria mellea* Isolate MEX100 5.8S ribosomal RNA Gene Sequence; Internal Transcribed Spacer 2, Complete Sequence; and 28S Ribosomal RNA Gene Sequence	0	379
Auxin-Responsive Protein IAA32	*CeIAA32*	TR21282|c0_g1_i1	IAA32_ARATHRecName: Full = Auxin-Responsive Protein IAA32; AltName: Full = Indoleacetic Acid-Induced Protein 32	5.54 × 10^−52^	*N attenuata* Auxin- Responsive Protein IAA32-like (LOC109235840), mRNA	1.44 × 10^−64^	861
Agamous-like MADS-Box Protein AGL65-like	*CeAGL65-like*	TR7267|c0_g1_i3	AGL65_ARATHRecName: Full = Agamous-like MADS-Box Protein AGL65	2.14 × 10^−38^	*N. tomentosiformis* Agamous-like MADS-Box Protein AGL65 (LOC104108062) Transcript Variant X2, mRNA	1.62 × 10^−42^	1827
Indole-3-acetic Acid-Amido Synthetase GH3.6	*CeGH3.6*	TR39668_c4_g1_i1	GH36_ARATHRecName: Full = Indole-3-acetic Acid-Amido Synthetase GH3.6; AltName: Full = Auxin-Responsive GH3-like Protein 6; Short = AtGH3-6; AltName: Full = Protein DWARF IN LIGHT 1; Short = DFL-1	4.22 × 10^−108^	*Ricinus communis* Indole-3-acetic Acid-Amido Synthetase GH3.6 (LOC8287873), mRNA	2.74 × 10^−61^	701
Probable Indole-3-pyruvate Monooxygenase YUCCA7	*CeYUC7*	TR46165|c0_g1_i1	YUC7_ARATHRecName: Full = Probable Indole-3-pyruvate Monooxygenase YUCCA7; AltName: Full = Flavin-containing Monooxygenase YUCCA7	2.41 × 10^−34^	*Ipomoea nil* Probable Indole-3-pyruvate Monooxygenase YUCCA7 (LOC109161643), mRNA	7.52 × 10^−42^	231
Somatic Embryogenesis Receptor Kinase 1	*CeSERK1*	TR38192|c1_g2_i6	SERK1_ARATHRecName: Full = Somatic Embryogenesis Receptor Kinase 1; Short = AtSERK1; AltName: Full = Somatic Embryogenesis Receptor-like Kinase 1;	2.97 × 10^−23^	*Rosa chinensis* MDIS1- Interacting Receptor like Kinase 1-like (LOC112177057), mRNA	1.36 × 10^−10^	438
Protein with Unknown Function 1	*CeNA1*	TR23240|c0_g1_i1	NA	NA	NA	NA	725
Protein with Unknown Function 2	*CeNA2*	TR48752|c0_g1_i1	NA	NA	NA	NA	441
Protein with Unknown Function 4	*CeNA4*	TR1001|c0_g1_i1	NA	NA	NA	NA	274

**Table 3:** Samples of nine centaury tissues and organs in which the expression of genes with a potential role in the SE was evaluated.

Abbreviation	Tissue Sample	Conditions
rl	Rosette Leaf	In Vitro; PGR-Free MS Medium; In Light
rr	Rosette Root	In Vitro; PGR-Free MS Medium; In Light
nl	Leaf from Flowering Plant	Nature
nr	Root from Flowering Plant	Nature
oc	Organogenic Callus	In Vitro; Induced on Leaf Explants on MS Medium Enriched with 0.2 mgL^−1^ 2,4-D and 0.5 mgL^−1^ CPPU; In Light
abl	Adventitious Bud	In Vitro; Induced on Leaf Explants on MS Medium with 0.2 mgL^−1^ 2,4-D and 0.5 mgL^−1^ CPPU; In Light
ec	Embryogenic Callus	In Vitro; Induced on Leaf Explants on MS Medium with the Addition of 0.2 mgL^−1^ 2,4-D and 0.5 mgL^−1^ CPPU; In Darkness
gse	Globular Somatic Embryo	In Vitro; Induced on Leaf Explants on MS Medium With 0.2 mgL^−1^ 2,4-D and 0.5 mgL^−1^ CPPU; In Darkness
cse	Cotyledonary Somatic Embryo	In Vitro; Induced on Leaf Explants on MS Medium with 0.2 mgL^−1^ 2,4-D and 0.5 mgL^−1^ CPPU; In Darkness

## Data Availability

The sets of transcriptome-based selection of differentially expressed genes during organogenesis and somatic embryogenesis in *Centaurium erythraea* Rafn. are openly available in RADaR—Digital Repository of Archived Publications Institute for Biological Research “Sinisa Stankovic” at https://hdl.handle.net/21.15107/rcub_ibiss_7120).

## Supplementary Materials

The following supporting information can be downloaded at: https://www.mdpi.com/article/10.3390/ijms252413531/s1.
