# Supplementary material for: Transcriptome and Gene Expression Analysis Revealed CeNA1: A Potential New Marker for Somatic Embryogenesis in Common Centaury (Centaurium erythraea Rafn.)
Source: Int J Mol Sci. 2024 Dec 18;25(24):13531. doi: 10.3390/ijms252413531 (PMC11677695; doi:10.3390/ijms252413531)
Supplement: Supplementary file 1 [file ijms-25-13531-s001.zip › ijms-3340973-supplementary.pdf]

Table S1. Primer sequences of selected candidates for evaluating gene expression with a potential role in SE in a collection of centaury tissues. The table shows transcript IDs for the selected isoforms on whose sequences the primers were designed, the annealing temperature of primers with the template (Ta), amplicon length, and RT-qPCR reaction efficiency (Eff).

**Gene product/gene**		**Trinity transcript ID**	**Primer sequence 5 → 3**	**Ta (°C)**	**Amplicon length (bp)**	**Eff (%)**
**Major allergen Pru ar 1**	*CePR10*	TR28332|c0_g4_i1	F - CTCATCCACCCTCTGCTTCG<br>R – ATCACTTCCCCAATCTCAGC	56.5	195	97.54
**14 kDa proline-rich protein DC2.15-like**	*CeDC2.15-like*	TR12274|c0_g1_i1	F - ATCCCAACGACAGCACCA<br>R – AGGGCTTGAGAGATGGCTTC	54	173	87.65
**Glycine-rich protein 3-like**	*CeGRP3-like*	TR3877|c0_g1_i1	F - AGTCCACAGTTTTATTCTGCTCT<br>R – CAGGTGCTGCTACAATGCTAA	56.5	161	95.60
**Desiccation-related protein PCC13-62-like**	*CePCC13-62-like*	TR30412|c0_g1_i1	F - CCTTCGTCCTTGAGTCCACAT<br>R – TGGCACAAACCCCTTCCTTATT	58.5	213	93.67
**ACS synthase 3**	*CeACS3*	TR36872|c0_g1_i2	F - GAGTCTTGCCCATGAGAATTGC<br>R – TCTCGGGGTGGAGGATTGC	58.5	227	92.61
**Thaumatin-like protein 1**	*CeTLP1-like*	TR30219|c0_g1_i1	F - CGTAAGAAAAAGAATGGCAGCAG<br>R – CCTCTTTGAGTTCTTCACTTGGA	58.5	110	88.76
**Tryptophan aminotransferase 1**	*CeTAR1*	TR20674|c0_g3_i1	F – ATGCCTTGGTAGTCCGTGTC<br>R – GCGTTTGCTGTCCGAGTTG	57.8	122	87.89
Continuation of Table S1. on the next page.<br>Table S1. (continued)						
**Agamous-like MADS-box protein AGL65-like**	*CeAGL65-like*	TR7267|c0_g1_i3	F - ATAATACGATGCTTGTCCGCC<br>R - AGAACCGTGATTGCTCCTAC	56.5	185	104.43
**Auxin-responsive protein IAA32**	*CeIAA32*	TR21282|c0_g1_i1	F - TTGAACAGTGCCGATGGAGA<br>R – TGACGAGCAGTGGAGAATGG	58.5	120	94.62

**Probable indole-3-pyruvate monooxygenase YUCCA7**	*CeYUC7*	TR46165|c0_g1_i1	F - AGGCATCACTTTCATCATTGTTACC<br>R - CAGGGGAGGCATTTGAAGGAA	58.5	194	95.68
**Somatic embryogenesis receptor kinase 1**	*CeSERK1*	TR38192|c1_g2_i6	F - AGTTGATTTGAGGAAGCGTCT<br>R - TTCCCTTTTGCTTCTCTGTCG	56.5	272	88.09
**Protein with unknown function 1**	*CeNA1*	TR23240|c0_g1_i1	F - TTAGAGCCACCACAGTCG<br>R - GGTCTTTCCAGAGTGTCATCA	55.1	199	91.40
**Protein with unknown function 2**	*CeNA2*	TR48752|c0_g1_i1	F - GCAGGTCTCCTTTAGTTCCG<br>R - GCTGTTGGCAGGGATTTTCA	57.1	120	96.75
**Protein with unknown function 4**	*CeNA4*	TR1001|c0_g1_i1	F - TTCTACCTCTTCTCACACCATCA<br>R - GAGACAGGGGAAACGAGC	56.5	101	96.74
**Indole-3-acetic acid-amido synthetase GH3.6**	*CeGH3.6*	TR39668_c4_g1_i1	F - TGCTTTGTGGGCTTTGCTTG<br>R - TCCCTGTTCTGATGTCCTTGC	59.1	129	92.71
